# Indian scenario of IgA nephropathy: a systematic review and meta-analysis

**DOI:** 10.4314/ahs.v21i1.21

**Published:** 2021-03

**Authors:** Anju Khairwa

**Affiliations:** Departments of Pathology, ESIC Model Hospital, Gurugram, India

**Keywords:** IgA Nephropathy, histomorphology, prevalence, India

## Abstract

**Background:**

IgA nephropathy (IgAN) is most common primary glomerulopathy. There are variations in prevalence of IgAN and its clinical features in different studies from India.

**Aim:**

To summarize overall scenario of IgAN in India.

**Methods:**

In this systematic review, studies related to IgAN and related renal disease were included. Data searched were PubMed, EMBASE, Google scholar, and Cochrane Database from inception to 31st January 2019

**Results:**

Total 49 studies (N=2480) were included: 21 studies (N=2309) of primary IgAN; 19 studies (N=21) of Secondary IgAN; four studies (N=133) of IgA vasculitis nephropathy (IgAVN); and five studies (N=17) of IgA dominant nephropathy (IgADN). Prevalence of IgAN was 16.5% in India. Age of affected persons was ranging from 27.2±16.7 to 48.6±21.3 years . Male female ratio was 1.8:1. Clinical features of Primary IgAN, IgAVN, IgADN & Secondary IgAN were microscopic hematuria (49.6%, 44.4%, 15.6% & 59.5%), macroscopic hematuria (5.1%, 0.4%,40.9%,& 35.7%), Subnephrotic proteinuria (42.1%, 29.4%, 23.2%, & 52.3%), nephrotic proteinuria (16.0%, 4.4%, 76.8%,& 47.6%), and hypertension (25.8%,18.3%, 35.5%,& 47.6%).. The 24 hours proteinuria was ranging from 2.6±1.5 to 4.7±2.3 gm/day and serum creatinine (mg/dl) was ranging from 0.9±0 to 3.5±3.9 mg/dl. Histolomorphologically, all type of IgAN showed mesangial hypercellularity and Immunofluorescence revealed IgA deposition..

**Conclusion:**

The overall prevalence of primary IgAN in India was 16.5%. The subnephrotic proteinuria and microscopic hematuria were common clinical features.

## Introduction

IgA nephropathy (IgAN) was first demonstrated by Berger in 1768.^1^ It is characterized by persistent microscopic hematuria, sub-nephrotic proteinuria, episodic gross hematuria, normal to severe impairment of renal function and hypertension.^1^,^2^

Histomorphology varies from normal to chronic glomerulonephritis on renal biopsy of these patients. The most common histomorphology is focal glomerulonephritis. 1,2 On immunofluorescence microscopy (IF), all cases had mesangial deposition of IgA dominantly and less commonly weak staining for IgG and C3. Theelectron microscopy (EM) had presence of mesangial immune complex deposits.^1^,^2^ IgA nephropathy is most common primary glomerulonephritis world-wide.^3^,^4^ IgA nephropathy mostly diagnosed in certain Asian countries, e.g. Japan, Mainland China, and Singapore.^5^,^6^ The incidence of IgA nephropathy ranges from 4.0% to 35.5% in the world.^7^ IgA nephropathy occurs in all age groups from children to elderly peoples, but it mostly affected age group is 10 to 40 years..^8^ Clinically features of patients with IgA nephropathy include microscopic hematuria or gross hematuria after one to two days or at a time of fever or upper respiratory tract infection (sore throat), but occasionally it may be associated with gastroenteritis, pneumonia, or urinary tract infection.^9^ It may remain asymptomatic and diagnosed incidentally or during routine screening of urine.^9^ The incidence and prevalence of IgA nephropathy is varied in different studies from India.^7^ Aim of index study was to evaluate the scenario of IgA nephropathy in India.

## Methods

We performed a systematic review and meta-analysis to assess the overall scenario of IgAN in India. We included studies of IgA nephropathy and IgA related renal diseases reported from India. Type of studies included were observational studies. We excluded randomized controlled trials and studies from outside of India. Studies including both children and adults were eligible for inclusion in the review. Renal biopsy was considered as reference standard. We searched PubMed (inception to 31^st^ January 2019), EMBASE (inception to 31^st^ January 2019), Google scholar (inception to 31^st^ January 2019) and Cochrane Database Reviews on 31^st^ January 2019. Search strategy for PubMed included (IgA nephropathy) OR IgAN)). We searched EMBASE with key words /exp IgA GN OR IgA nephropathy AND (IgAN), Google scholar with IgA GN or IgA nephropathy and Cochrane Database Reviews with IgA nephropathy. References of included studies were hand searched for additional studies. We assessed the studies for inclusion and extracted data for review.

Primary outcome of review was prevalence of IgA nephropathy in India. Secondary outcomes included clinical features, laboratory findings, histopathological pattern, immunofluorescences findings and electron microscopic findings of IgAN from India in comparison to foreign studies.

## Statistical methods

The data were entered in excel sheet and analyzed using STATA 12.0 and Cochrane Rev Man 5.1. Continuous outcome data were reported as mean and dichotomous data as percentages.

The Newcastle-Ottawa Scale (NOS) was used for assessing quality of nonrandomized studies (cohort and case-control studies).^10^

## Results

**Figure 1:**
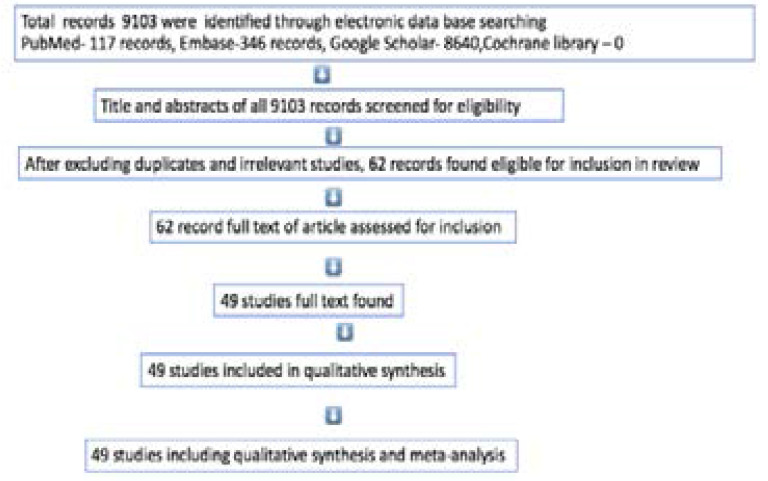
Study selection flow diagram

The study selection flow diagram is shown in Figure 1. By electronic database search we found a total of 9103 records and screened titles and/or abstracts of all records. After excluding duplicates and irrelevant studies, 62 eligible studies were assessed for full texts. Full texts were found for 49 studies and these were including for qualitative synthesis and meta-analysis.

**Figure 2:**
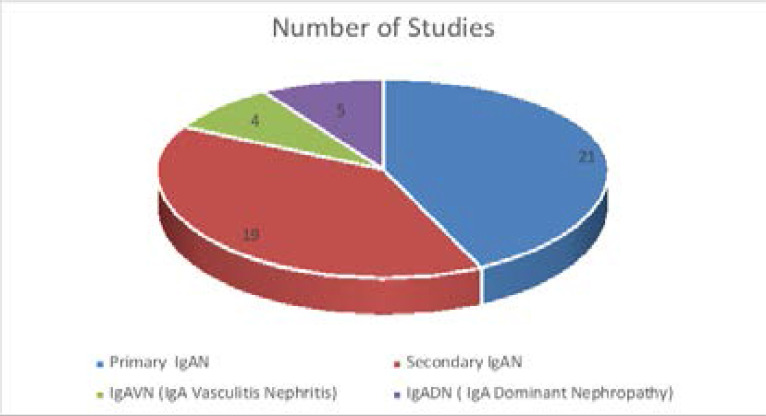
Subgrouping of the 49 studies in four

Out of 49 studies, 21 studies (2309 cases) were related to Primary IgAN, 19 studies (21 cases) were related to secondary IgA nephropathy (Secondary IgAN), four studies (133 cases) were related to Henoch-Schönlein purpura (HSP) with IgA nephropathy or IgA vasculitis nephritis (IgAVN), and five studies (17 cases) were related to IgA dominant nephropathy (IgADN) (figure 2). The frequency/ prevalence of IgA nephropathy was reported in 14 studies and is shown in Table 1.

**Table 1:** Frequencies/prevalence (%) of IgA nephropathy in India

S. No.	Reference	Year	Study population Children, Adult	Prevalence of IgA N (%)
1.	Chacko et al	2011	C, A	8.6
2	Ganesh et al	2018	C, A	21.6
3	Siddappa S	2011	A	7.8
4	Das et al	2015	A, C	7.5
5	Agrawal et al	2017	A	2.6
6	Mittal et al	2012	A	8.1
7	Chandrika et al	2009	A, C	14.2
8	Ramakrishan	2016	A	11.5
9	Chacko et al,	2007	A,C	32
10	Vanikar et al,	2005	A	16.5
11.	Bhuyan et al	1992, 87	A,C	7.24
12.	Tiwari et al	2016	A,C	14.3
13	Chowdary et al	2018	A	42.0
14	Dhanapriya et al	2018	A	5
After pooling the data total prevalence/frequencies				**16.5%**
C-children, A-Adult,				

Clinical and laboratory features of Primary IgA nephropathy, IgA vasculitis nephritis (HSP), IgA dominant nephritis and Secondary IgAN are shown in Table 2. Histomorphology (MEST-C& Haas's classification), Immunofluorescences, and Electron microscopic findings of Primary IgAN, IgAVN (HSP), IgADN and Secondary IgAN are described in Table 3.

**Table 2:** Clinical and Laboratory features of Primary IgA nephropathy, IgA vasculitis nephritis (HSP), IgA dominant nephritis and Secondary IgAN

S.No.	Main clinical features	Primary IgA nephropathy	IgA vasculitis nephritis(HSP)	IgA dominant nephritis	Secondary IgAN
1.	Mean age (years)	31.7±13.8	37.5±15.8	48.6±21.3	27.2±16.7
2.	Male : female	1.93:1	1.07:1	1.64:1	2:1
3.	Asymptomatic urinary abnormalities (%)	5.5	1.4	0	9.5
4.	Macroscopic hematuria(%)	5.1	0.44	40.94	35.71
5.	Microscopic hematuria(%)	49.6	44.49	15.64	59.57
6.	Subnephrotic proteinuria (>1g/d) (%)	42.1	29.42	23.29	52.38
7	Nephrotic range proteinuria (>3g/d) (%)	16.04	4.44	76.88	47.61
8.	Loin or abdominal pain(%)	1.8	61.72	0	4.7
9.	Hypertension(%)	25.8	18.39	35.5	47.61
10.	Infection-related exacerbations(%)	10.3	19.14	47.05	4.7
11.	Skin Rashes(%)	0	99.24	0	0
12.	GIT manifestation(%)	0	54.83	5.8	0
13.	Arthritis/arthralgia(%)	0	78.58	0	0
14.	Duration of symptoms	5.40±3.2	3.91±0.59	2.87±1.84	2.81±3.9
15.	Raised serum creatinine	3.04±2.6	0.9±0	3.21±20	3.5±3.9
16.	Reduced e-GFR	59.54±19.3	-	63.6±0	78±0
17.	24 h urine protein (g/d)	2.6±1.5	3.2±0	3.5±0.2	4.7±2.3
18.	Serum C3 level reduced (%)	0	0.06	88.47	0

**Table 3:** Histomorphology (MEST & Haas's classification, Immunofluorescences and Electron microscopic findings in primary IgA nephropathy, IgA vasculitis nephritis (HSP), IgA dominant nephritis and Secondary IgAN

S.No.	Types IgAN	Investigations											
		Histomorphology(%)			Immunofluorescence (%)					Electron microscopy(%)			
		MEST-C classification	Haas's classification	Skin biopsy	IgG	IgA	IgM	C3	F.H.	MeD	SubEnD	SubEpD	Both
		Renal biopsy											
1.	Primay IgAN	Me-**59** En-10.4 SG-**20.9** C1–3.6 C2–5.3 T0-2.8 T1-**11.5** T2-9.6 N-1.03	Cl I -11.1 Cl II-10.6 Cl III-13 Cl IV-7.5 Cl V-**16.2**	-	1.6	**100**	4.5	4.6	0.2	**26.4**	0.58	0	**17.3**
2.	IgAVN	Me-**9.7** En-6.7 SG-0.7 C1-2.2 T0-2.2	-	LC-**33.8**	0	**100**	0	**22.5**	0	-	-	-	-
3.	IgADN	Me-29.4 En-47.0 C2-**58.8**	-	-	12.2	100	1	**89.7**	0	0	5.8	**11.7**	5.8
4.	Secondary IgAN	Me-**95.2** En-23.8 SG-33.3 C1-19 T0-14.2	-	-	16.6	**100**	5.9	**9.5**	0	9.5	0	4.7	0

Me-Mesangial proliferation, En-Endocapillary proliferation, SG-Segmental Glomerulosclerosis, C1- <25% Cellular/fibrocellular crescent, C2- >25%Cellular/Fibrocellular crescent, T0- ≤25%TA/IF T1- 26% to 50% TA/IF, T2->50%TA/IF, N-Normal, Cl- Class, MeD-mesangial deposition, SubEnD-subenothelial deposition, SubepD-subepithelial deposition, Both-MeD+EnD, ,HistoM-histomorphology, LC- leukocytoclastic vasculitis ,F.H.-Full House

## Discussion

We found combined prevalence of IgAN as 16.5% of in India. The prevalence of IgA nephropathy showed considerable variations (0.8–47%) among geographic regions with different renal biopsy practices.^3^,^6^,^25^–^26^ The prevalence in India was lower than certain Asian counties e.g. Japan (47.4%) and China(45%).^25^–^26^ We found higher prevalence of IgAN in comparison to study by Seedate et al (13.3%) from African country Natal done on Indians.^27^ The IgA nephropathy was uncommon (0.8%) in African blacks.^27^ The frequencies of IgAN was higher in India than the studies from United States that had prevalence of 10.8% and 9.4%.^28^ We were unable to pool the data for incidence because the studies were not accurately defined the incidence. Primary IgAN and Secondary IgAN was found in younger age groups and it is similarly reported by other studies^29^,^30^ Males were more commonly effected than females and our findings corroborated with other studies^27^,^29^ We found that IgADN was presented in higher age group (48.6±21.3) than Primary IgAN, IgAVN, and Secondary IgAN. Similarly, Nasr et al reported IgADN in elderly age group.^31^ We also found rapid onset of symptoms in primary IgAN and Secondary IgAN as described by other studies.^9^,^32^ It was different from PIGN (post infection glomerulonephritis), which had a period of latency (1–2 weeks) after the symptoms of infection.^33^ Infection related exacerbations were associated maximum with IgADN (47%) followed by IgAVN (19%). The similar observation was reported in a study by Satoskar et al.^34^ Episodic macroscopic hematria, nephrotic range proteinuria and hypertension had been typically associated with IgADN.^35^ We found the similar observations. Microscopic hematuria (49.6–59.5%) and subnephrotic proteinuria (42.1–52.3%) were more frequent in primary and secondary IgAN and it correlated with other studies.^36^–^38^ We found that rapidly progressive renal failure was associated with IgADN and secondary IgAN than primary IgAN and IgAVN and similar trend had been demonstrated by other studies.^35^,^37^ Similar to Nasr et al., we also found hypocomplementemia more in IgADN in comparison to primary and secondary IgAN and IgAVN.^39^ Histomorphology of primary and secondary IgAN ranged from normal to chronic glomerulonephritis and it was similar to various other studies.^29^, ^36^, ^40^,^41^ On IF, most biopsies showed predominantly IgA deposition in primary and secondary IgAN at mesangial region and same had been demonstrated by many studies.^42^–^44^ Electron microscopy depicted mesangial electron-dense deposition followed by both (mesangial and subendothelial) deposition in primary and secondary IgAN, and similar findings were reported by Haas et al.^45^ IgAVN (HSP) showed highly variable histomorphology of glomeruli ranging from normal to diffuse proliferative and crescentic glomerulonephritis. 46 Skin biopsy demonstrated leukocytoclastic vasculitis in (33.8%) cases, which was less than other study.^47^

The limitation of this study was that we were unable to pool incidence of IgAN because none of studies clearly defined the data.

## Conclusion

The IgA nephropathy was more prevalent in India with most common subgroup being primary IgAN. Subnephrotic proteinuria and microscopic hematuria were common clinical presentation of IgAN. Secondary IgAN and IgADN had more rapidly-progressive renal failure than primary IgAN and IgAVN.
